# Evolutionary Dynamics of Matrix Metalloproteases with Collagenolytic Activity in Teleosts

**DOI:** 10.3390/ani15223270

**Published:** 2025-11-12

**Authors:** Rafael Angelakopoulos, Andreas Tsipourlianos, Ioannis Damianos Maravelakis, Themistoklis Giannoulis, Zissis Mamuris, Katerina A. Moutou

**Affiliations:** 1 Laboratory of Genetics, Comparative and Evolutionary Biology, Department of Biochemistry and Biotechnology, University of Thessaly, 41500 Larissa, Greece; rangelak@uth.gr (R.A.); antsipou@uth.gr (A.T.); ianos209m@gmail.com (I.D.M.); zmamur@uth.gr (Z.M.); 2 Laboratory of Biology, Genetics and Bioinformatics, Department of Animal Science, University of Thessaly, 41334 Larissa, Greece; thgianno@uth.gr

**Keywords:** matrix metalloproteases (MMPs), whole-genome duplication, teleost evolution, regulatory sub-functionalization

## Abstract

Matrix metalloproteases (MMPs) are zinc-dependent endopeptidases that are critical for extracellular matrix (ECM) remodeling, with key roles in tissue development and repair, and immune responses. Despite their evolutionary and functional importance, the diversification and regulatory dynamics of MMPs genes in teleosts remain poorly understood. This study investigates the evolutionary history of MMPs with collagenolytic activity in the vertebrates with an emphasis on teleosts. Using comparative genomics and phylogenetics we identified conserved single-copy *mmp2* and *mmp9* genes and duplicated *mmp11* and *mmp13* paralogs in all non-salmonid teleosts. Phylogenetic and synteny analyses suggest that *mmp11* paralogs originated from the teleost-specific genome duplication (TSGD), whereas the origin of *mmp13* paralogs originated from a more complex evolutionary scenario. Protein domain analysis confirmed conserved catalytic motifs across species, supporting functional retention. The expression patterns of paralog genes were studied in two model marine teleosts, the *Sparus aurata* (gilthead sea bream) and *Dicentrarchus labrax* (European sea bass). Developmental and adult tissue transcriptome analyses revealed three major expression patterns among the paralogs: similar expression, stage-specific or tissue-specific expression. The overall data point to varied evolutionary dynamics of *MMP* genes in the teleosts, tracing their origin to different whole-genome duplication events. Expression profiles on paralog genes in model teleosts suggest regulatory sub-functionalization as the most possible fate of retained MMPs paralogs in teleosts following whole-genome duplication.

## Simple Summary

Fish, like all animals, need enzymes that can break down and rebuild the material surrounding their cells. These enzymes, called matrix metalloproteases, are essential for growth, tissue repair, and defense against disease. Despite their importance, we know little about how the genes behind these enzymes evolved in fish, which make up the largest and most diverse group of vertebrates. In this study, we traced the history of the genes that produce collagen-degrading metalloproteases across different animals, with a focus on fish. We discovered that while some of these genes remain single copies, others were duplicated during fish evolution and kept their function. By studying two important farmed fish, *Sparus aurata* (gilthead sea bream) and *Dicentrarchus labrax* (European sea bass), we found that the duplicated genes are regulated differently at different developmental stages or in different tissues. Our findings provide new insights into the biology and evolution of fish and may help improve aquaculture practices by linking gene function to fish development and health.

## 1. Introduction

Extracellular matrix (ECM) consists of a complex network of macromolecules, providing structural stability and support to the cells [1]. ECM acts essentially as a “scaffold”, facilitating cell adhesion and migration [2]. Proteins such as collagen provide structural integrity, and glycoproteins, such as fibronectin and laminin, promote cell adhesion. In addition, proteoglycans play a vital role in stability by contributing to viscosity and compressive resistance. Collagen is the most abundant protein in ECM and it is characterized by a triple-helix conformation. Key biological processes, namely embryogenesis, tissue remodeling, osteogenesis, and angiogenesis require collagen degradation performed by collagenolytic enzymes such as matrix metalloproteases (MMPs) [3,4,5].

Matrix metalloproteases (MMPs) are a family of zinc-dependent endopeptidases, playing a key role in the regulation of ECM remodeling, tissue growth and homeostasis in general. They belong to the metzincin superfamily characterized by a conserved zinc-binding motif (metzincin motif-HEXXHXXGXXH) where the three histidine residues are coordinated with a zinc ion, essential for MMPs catalytic activity [6]. These proteases are present in all kingdoms of life, and they are found in both vertebrates and invertebrates [7,8]. Phylogenetic studies suggest that MMP vertebrate gene family was expanded from a common protostome-deuterostome ancestor through multiple rounds of genome duplications. However, prior to the emergence of vertebrates the family included a low number of gene members (2 MMPs in protostomes and 5 MMPs in urochordates). Interestingly, although teleosts have undergone an extra round of genome duplication compared to other vertebrates, they possess a similar number of genes to the other vertebrates, with some of the family members missing [6].

Whole-genome duplication (WGD) events have been a recurring phenomenon throughout the evolutionary history of vertebrates and other eukaryotes contributing to the long-term evolutionary success of species-rich lineages [9,10]. Unlike duplication of individual genes or small gene clusters, WGD events stand out to facilitate the divergence of entire networks of ohnologues, driving molecular and phenotypic complexity [11]. Ohnologs or paralogs are gene duplicates that originate from whole-genome duplication events and are named after Susumu Ohno, who first proposed their evolutionary importance. Vertebrates have undergone two shared whole-genome duplication events (1R and 2R) [12] and teleosts, after their diversification as a lineage, have undergone a third WGD event (3R or TS-WGD) 320–350 Mya [13,14]. After a WGD event, the trajectory of duplicated genes is directly dependent on evolutionary pressures [15]. Because both gene copies can initially perform the same function, one copy can accumulate mutations without compromising organismal fitness. This functional redundancy temporarily relaxes purifying selection, making ohnologs less subject to evolutionary constraints and allowing sequence and regulatory divergence to occur. Duplicated genes are free of evolutionary constraints, creating fertile ground for the accumulation and establishment of mutations [16]. This can lead to a functional loss of one of the two copies, creating pseudogenes, provided the gene is not under evolutionary pressure. On the other hand, the maintenance of paralogs genes, either by acquiring a new function or by diverging the function of one, is driven by the advantages of duplicating a genetic locus under selection [17]. These mechanisms can act sequentially or simultaneously during gene evolution, contributing to the differential fate of paralogs genes. Ultimately, the establishment of duplicated gene loci depends on several factors, such as the presence of neutral or deleterious mutations, selection factors, and, in some cases, population size [18].

Progress in unraveling the evolutionary history of various gene families has been achieved through the sequencing of complete teleost genomes. However, there is a noticeable lack of literature on matrix metalloproteinases (MMPs), a gene family whose evolution and transcriptional regulation remain poorly understood despite their significant relevance in many eukaryotic species. While insights into the evolution and transcriptional regulation of various gene families have been gained, the influence of these processes on the MMP gene family is still largely unknown [19].

Teleost fish species occupy a critical position in the vertebrate lineage, allowing us to explore the evolutionary dynamics of matrix metalloproteinases (MMPs) with collagenolytic activity, in the context of whole-genome duplications (2R and 3R events). *Sparus aurata* (gilthead seabream) and *Dicentrarchus labrax* (European seabass) serve as valuable models due to their phylogenetic relevance, conservation, and accessibility [20,21]. Studying MMPs in these species provides insights into the conservation and diversification of MMP functions within teleosts. Additionally, their accessibility and economic importance make gilthead sea bream (*S. aurata*) and European sea bass (*D. labrax*) feasible choices for experimental research, facilitating the collection of samples and the undertaking of analyses across various tissues and developmental stages. This unique combination of phylogenetic significance, conservation relevance, and experimental feasibility make these fish species ideal candidates for advancing our understanding of MMP biology in aquatic environments. In this study, we tested the hypothesis that the collagenolytic matrix metalloproteases (MMPs) paralogs in teleosts have undergone regulatory sub-functionalization following gene duplication, resulting in distinct expression patterns in development and tissue remodeling. Using gilthead sea bream (*S. aurata*) and European sea bass (*D. labrax*) as model species, we integrated comparative genomic and transcriptomic analyses to (i) trace the evolutionary divergence of *MMPs* paralogs, and (ii) evaluate their tissue- and stage-specific expression.

## 2. Materials and Methods

### 2.1. Phylogenetic Analysis

#### 2.1.1. Database Mining and Sequence Retrieval

A comprehensive database mining was performed to identify genes belonging to the Matrix Metalloproteases family, as described in the HGNC database for humans. Subsequently, for members of the gene family with collagenolytic activity, we utilized the BLASTP algorithm of the ENSEMBL database (ENSEMBL 105) [22] to retrieve homologous genes from twenty species. Specifically, sequences belonging to representatives of teleosts (13), a representative of *Actinopterygii*, representatives of *Sarcopterygii* (5), and a representative of *Chondrichthyes* were selected to elucidate the evolutionary path of the *MMP2*, *MMP9*, *MMP11*, and *MMP13* genes (Table 1). The taxonomic sampling strategy was designed to span key vertebrate lineages and to provide phylogenetic resolution across major evolutionary transitions.

Representatives of Sarcopterygii were included as outgroups that have only undergone the two early vertebrate whole-genome duplications (1R and 2R), allowing for comparisons against lineages that experienced additional genome duplication. The *holostei* species (*Lepisosteus oculatus*), a non-teleost actinopterygii, was selected because it diverged just prior to the teleost-specific genome duplication (TSGD) and can act as a closely related outgroup for identifying the duplication origin of teleost paralogs. Teleost species were sampled across major clades to ensure broad coverage of the TSGD-derived lineage, while the Chondrichthyes species (*Callorhinchus milii*) served as a more distant outgroup to root the phylogeny and distinguish ancient from lineage-specific duplication events. This sampling enabled inference of ohnolog retention patterns and duplication timing.

In addition, to examine exon–intron organization, the gene structures of all *mmps* of gilthead sea bream (*S. aurata*) and European sea bass (*D. labrax*) were visualized using the Gene Structure Display Server (GSDS 2.0) [23]. For each gene, the coding sequence (CDS) and the corresponding genomic DNA sequence were retrieved from Ensembl and aligned in GSDS to infer intron positions relative to exon boundaries (Supplementary Figures S1–S4).

#### 2.1.2. Sequence Alignments and Phylogenetic Analysis

The retrieved sequences were used for the reconstruction of the phylogenetic tree and sequences of low quality were removed. Multiple sequence alignment was performed using the MAFFT algorithm (version 7) [24] along with the GUIDANCE2 algorithm [25] to gain statistical confidence for each aligned site. The multiple alignment data were uploaded to MEGAX [26] and the best-fitting substitution model was identified for each gene based on the Bayesian Information Criterion (BIC). The selected models used for tree reconstruction are reported in the corresponding figure legends. Lastly, Maximum Likelihood (ML) algorithm was used, and 100 bootstrap repetitions were performed to statistically analyze the sequence branches. In addition to the ML algorithm, a tree using MrBayes (version 3.2.7) and Bayesian inference was used to gain statistical confidence for each branch of the tree, using the default parameters (two independent runs of 1 million generations, sampling every 100 generations, and applying a burn-in of 25%) [27].

#### 2.1.3. Collinearity and Synteny Analysis and Duplicate Gene Origin Classification

Phylogenetic analysis helps elucidate the evolutionary relationships among genes or species, providing insights into their common ancestry, divergence, and adaptation. But this type of analysis cannot answer questions about genomic regions with conserved architecture, which synteny can. This is pivotal for identifying orthologs and paralogs, helping to understand the evolutionary forces that shaped the genomic landscape.

Two different approaches were used to evaluate the conservation level of the genes of interest among the species studied. Firstly, collinearity analysis was performed using MCscanX (Multiple Collinearity Scan X, version 1.0.0), to examine the collinearity in the genomes of gilthead sea bream (*S. aurata*) and European sea bass (*D. labrax*). The detailed pipeline was according to Tsipourlianos et al., 2024 [28]. Secondly, to evaluate the conservation level of the adjacent gene environment for the genes of interest, the upstream and the downstream localization was examined for the absence or presence of a conservation level in the surrounding genomic area using BioMart tool of the ENSEMBL database [29]. Lastly, the MCScanX tool called duplicate gene classifier was used in order to classify duplicated genes according to their evolutionary origin [30].

#### 2.1.4. Functional Conservation Estimate

The Interpro database [31] was used to identify conserved regions in proteins of gilthead sea bream (*S. aurata*) and European sea bass (*D. labrax*) and compare with human homologous proteins. Then, the IBS (Illlustrator for Biological Sequences) Software (version 1.0) was used to visualize the protein domains and their functions [32].

### 2.2. Gene Expression Profiling

#### 2.2.1. Larvae Rearing and Sampling

Egg hatching and larval rearing for both European sea bass and gilthead sea bream were performed by using the methodology of Papandroulakis et al. at the Institute of Marine Biology, Biotechnology and Aquaculture (HCMR) [33]. Fish larvae were kept at 20 °C for gilthead sea bream (*S. aurata*) and 17 °C for European sea bass (*D. labrax*) from egg (epiboly) to metamorphosis stage. The same sampling protocol was followed for both species. Briefly, larvae samples were collected at three developmental stages, first feeding (FF); notochord flexion (FL); and mid-metamorphosis (MM), anesthetized using 2-Phenoxyethanol, rinsed with clean sea water and fixed in RNA later solution. Samples were stored at −20 °C until extraction [34].

#### 2.2.2. RNA Extraction and Transcriptome Sequencing

Larvae homogenization was performed through glass beads beating homogenization at 6000 rpm for 30 s (tissue homogenizer, Precellys, Bertin Technologies, Montigny-le-Bretonneux, France). To obtain sufficient total RNA, 3–5 individuals were pooled per sample at the FF stage, whereas individual extractions were performed at the FL and MM stages. Total RNA was extracted using E.Z.N.A.^®^ Total RNA Kit I (OMEGA bio-tek, Omega Bio-tek, Inc., Atlanta, GA, USA) with slight refinements. To totally remove traces of genomic DNA an extra step of DNAse treatment was performed with DNA-free™ DNA Removal Kit (Invitrogen, Waltham, MA, USA). Total RNA was evaluated via gel electrophoresis and was quantified using Qubit™ RNA BR Assay Kit (Invitrogen). Subsequently, twelve RNA samples were pooled, equimolarly for each developmental stage and sent to Novogene Inc. (Europe, Cambridge, UK), for transcriptome sequencing, using Novaseq 6000 Illumina’s platform (Illumina, San Diego, CA, USA) and 150 bp paired-end reads were generated producing approximately 30 million reads per sample, with 97% passing Q20. A pair of fastq files for each sample (R1: forward and R2: reverse) were acquired after the transcriptome sequencing and the bioinformatics analysis was performed according to Stamperna et al. and Robinson et al. [35,36]. The detailed pipeline can be found in the public repository Github (https://github.com/RafaelAngelakopoulos/Bioz_lab/tree/0f040a4aee3536952a6df587f25a02ddb74fa61b/RNAseq, accessed on 9 November 2025) and the transcriptome data are submitted in SRA (European sea bass (*D. labrax*): PRJNA1050410 and gilthead sea bream (*S. aurata*): PRJNA1050571).

In addition, publicly available transcriptome data (FASTQ files) from various adult tissues were obtained for both *gilthead sea bream* (*S. aurata*) and *European sea bass* (*D. labrax*) and processed with the same pipeline. Moreover, to account for potential batch effects in the transcriptomic data, we applied the ComBat-Seq model [37] from the sva R package (v3.56.0) [38]. The accession numbers of the data are shown in Supplementary Table S1.

## 3. Results

### 3.1. Phylogenetic Analysis

Using HGNC database, we identified *MMP1*, *MMP2*, *MMP9*, *MMP11* and *MMP13* genes as MMPs with collagenolytic activity. Gene names of fish paralogs and human homologs were inconsistent within Ensembl annotation, with some being marked as novel genes. Standardizing gene names was imperative to improve clarity throughout the manuscript. In cases Ensembl annotation lacked consistency in gene names, a nomenclature based on the human homolog (best BLAST hit) was adopted, appending an ascending number to each paralog. The correspondence between the masked gene names and the according Ensembl gene IDs is shown in Table 2.

**Figure 1:**
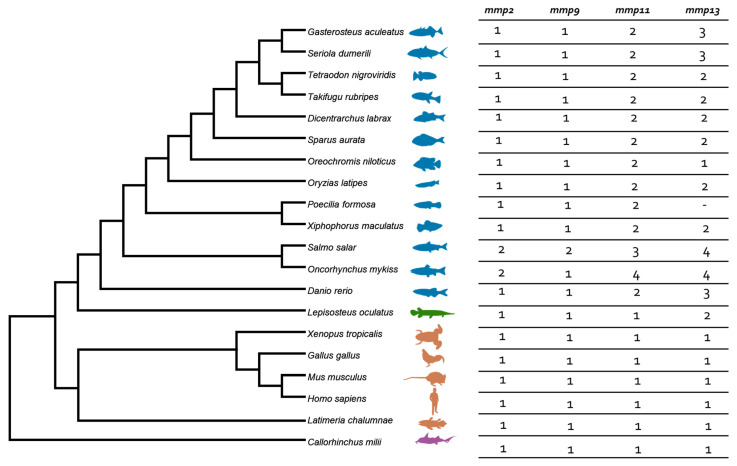
Species tree and summarized number of genes of *mmps* per species. Teleosts are presented in blue, *Lepisosteus oculatus* (*spotted gar*) in green, *sarcopterygii* in orange, and *C. milli* in purple. Colors are consistent in all gene trees.

Among the five genes initially identified within the human genome, we discovered an alternative repertoire in both gilthead sea bream (*S. aurata*) and European sea bass (*D. labrax*), each harboring a total of six genes. Interestingly, *mmp1* was not identified in teleost genomes. Duplications of *mmp13* and *mmp11* genes were recorded, while *mmp2* and *mmp9* harbored one copy each. The detailed number of genes in each species is summarized in Figure 1.

We used both Maximum Likelihood (ML) and Bayesian inference methodologies for tree reconstruction. Topologies of both methodologies were similar, so we integrated the results from both statistical models on the tree in a way of ML bootstraps/Bayesian probability.

**Figure 2:**
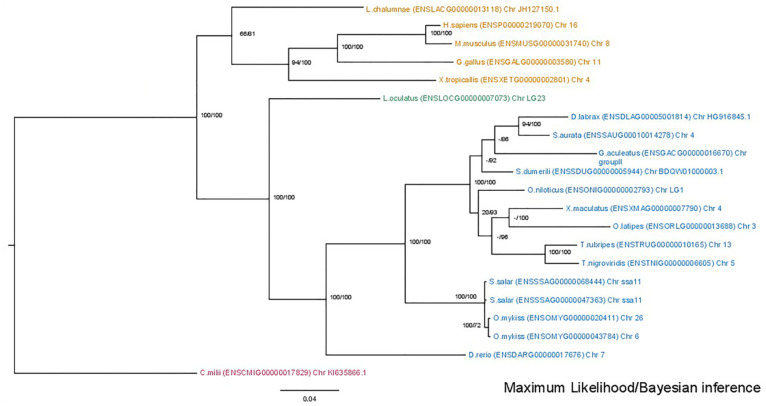
Phylogenetic tree of the mmp2 protein is depicted, with teleosts represented in blue, *Lepisosteus oculatus* (*spotted gar*) in green, *sarcopterygii* in orange, and *C. milli* in purple. Bootstraps from the Maximum Likelihood method are denoted on the right of “/”, while those from Bayesian inference are on the left. The LG + G replacement model was employed for reconstructing the ML tree.

**Figure 3:**
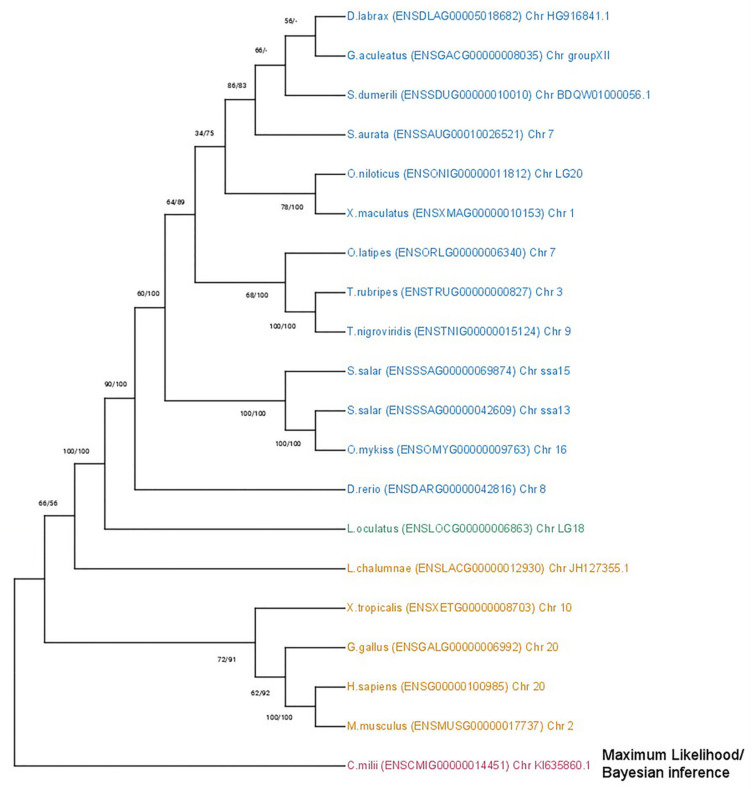
Phylogenetic tree of the mmp9 protein is depicted, with teleosts represented in blue, *Lepisosteus oculatus* (*spotted gar*) in green, *sarcopterygii* in orange, and *C. milli* in purple. Bootstraps from the Maximum Likelihood method are denoted on the right of “/”, while those from Bayesian inference are on the left. The LG + G replacement model was employed for reconstructing the ML tree.

The phylogenetic analysis of the *mmp2* gene (Figure 2) revealed a conserved single-copy presence across all species, except within the Salmonidae branch, where a duplication event is present, resulting in the retention of two gene copies. This observation aligns with the established fourth round of genome duplication specific to the Salmonidae lineage (4R). A similar topology was observed in the *mmp9* tree (Figure 3), wherein the gene maintained a single-copy status across diverse species, except for the Salmonidae branch, where gene duplication was evident. Notably, within this branch, only *Salmo salar* (salmon) exhibited the retention of paralogs derived from the 4R event, whereas *Oncorhynchus mykiss* (rainbow trout) retained a single copy of the *mmp9* gene, suggesting a probable loss of the second copy under evolutionary pressures.

**Figure 4:**
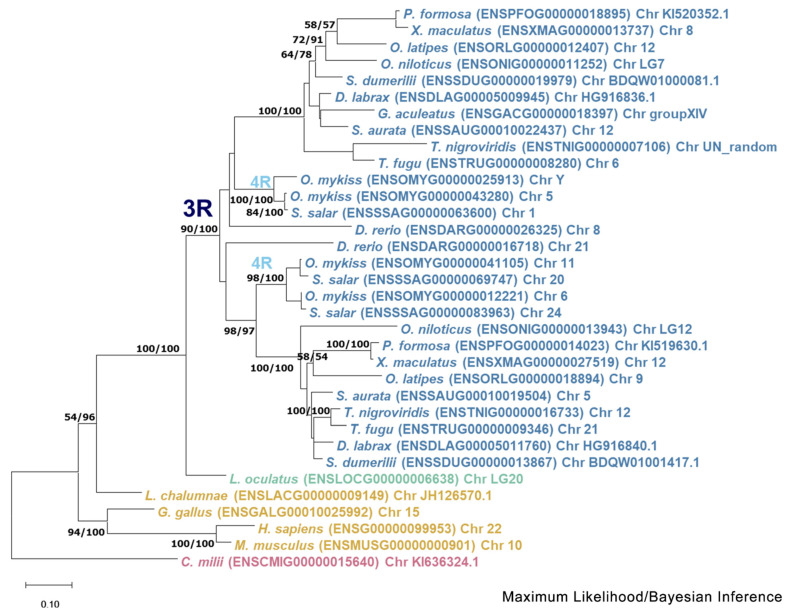
Phylogenetic tree of the mmp11 protein is depicted, with teleosts represented in blue, *Lepisosteus oculatus* (*spotted gar*) in green, *sarcopterygii* in orange, and *Callorhinchus milii* (*Elephant shark*) in purple. Bootstraps from the Maximum Likelihood method are denoted in the right of “/”, while those from Bayesian inference are on the left. The JTT + G + I replacement model was employed for reconstructing the ML tree. The main duplication events are annotated on the tree.

Regarding the duplicated genes *mmp11* and *mmp13* two topologies were discerned. The *mmp11* topology suggests that both paralogs originated from a whole-genome duplication event, most likely the TSGD, occurring in the common ancestor of teleosts (Figure 4). Most teleost representatives possess two copies of this gene, with the exception of the Salmonidae lineage, where four copies were identified. This inference is strongly supported by both the bootstrapping and Bayesian probabilities in the base of teleosts, splitting from spotted gar (*Lepisosteus oculatus*), a species that predated the TSGD. The tree is outgrouped by *Sarcopterygii*, although *Latimeria chalumnae* (Coelacanth) aligned with *Actinopterygii* albeit in a less supported branch. Lastly, the tree is rooted from *Callorhinchus milii* (Elephant shark) following the species tree topology.

**Figure 5:**
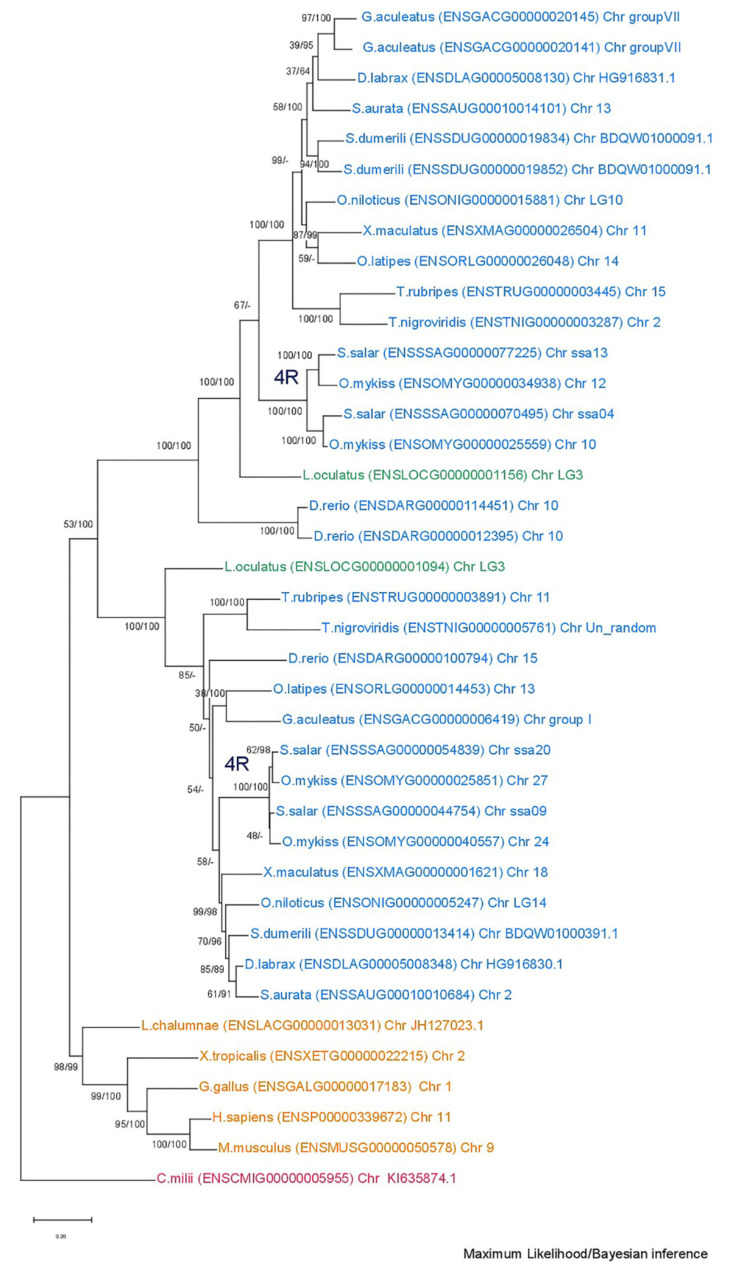
Phylogenetic tree of the mmp13 protein is depicted, with teleosts represented in blue, *Lepisosteus oculatus* (*spotted gar*) in green, *sarcopterygii* in orange, and *Callorhinchus milii* (*Elephant shark*) in purple. Bootstraps from the Maximum Likelihood method are denoted on the right of “/”, while those from Bayesian inference are on the left. The JTT + G + I replacement model was employed for reconstructing the ML tree. Main duplication events are annotated on the tree.

Phylogenetic analysis of the *mmp13* gene (Figure 5) reveals a discernible divergence of *Actinopterygii* from *Sarcopterygii*. The inferred topology does not provide enough clarity to confidently hypothesize the evolutionary scenario leading to the paralogs. Specifically, the tree configuration places two copies of the spotted gar gene across both teleost clades, with *Sarcopterygii* forming an outgroup. The placement of *Danio rerio* (zebrafish) outside of the spotted gar (*Lepisosteus oculatus*) suggests that the branching order of these species is not well-resolved, further complicating the interpretation of the tree.

**Figure 6:**
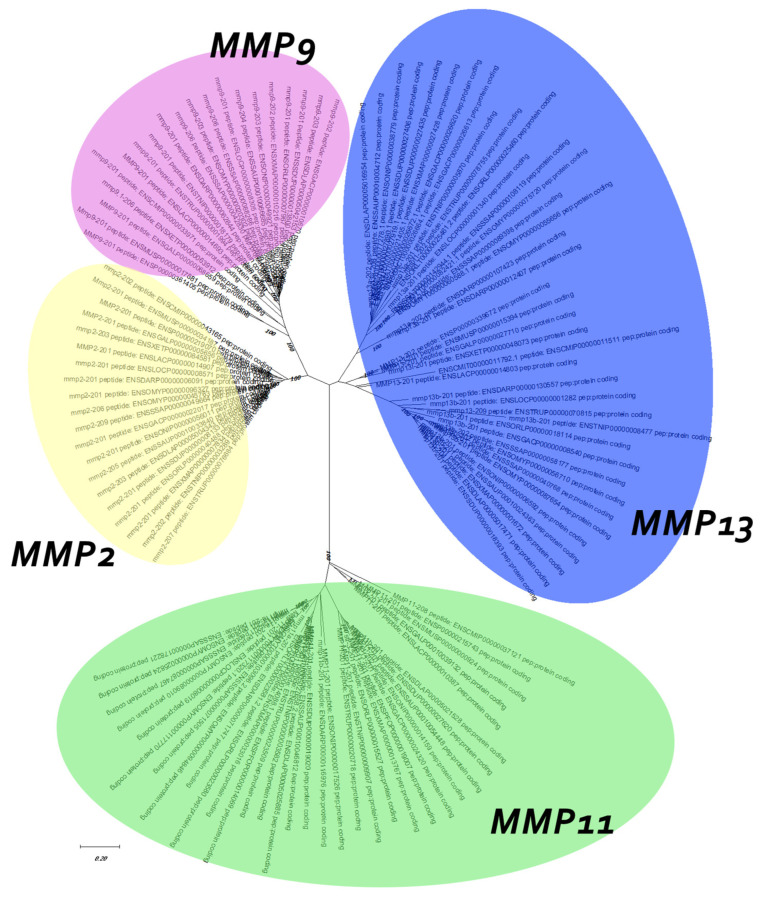
Unrooted family tree of MMPs under study. Model used for the ML tree: WAG + G + I.

Finaly, for better understanding the evolutionary relationship between the genes in study, a family unrooted tree was reconstructed using the ML algorithm. The topology revealed suggests that *mmp2* and *mmp9*, the two gelatinases, are more closely related than *mmp13* and *mmp11*, with the latter being the most diverged (Figure 6).

### 3.2. Synteny Analysis

**Figure 7:**
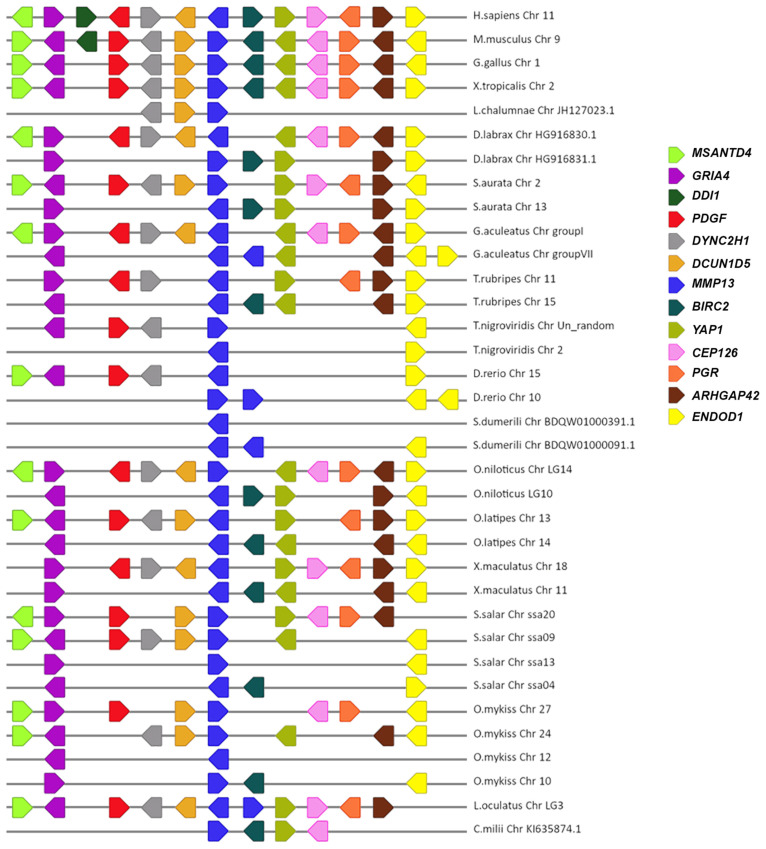
The micro-synteny of *mmp13* is visually depicted in the figure, where colored arrows denote homologous genes, and the arrowhead signifies the direction of predicted gene transcription. Horizontal lines represent chromosome fragments from the included species. Distances between genes or gene length are not drawn to scale.

**Figure 8:**
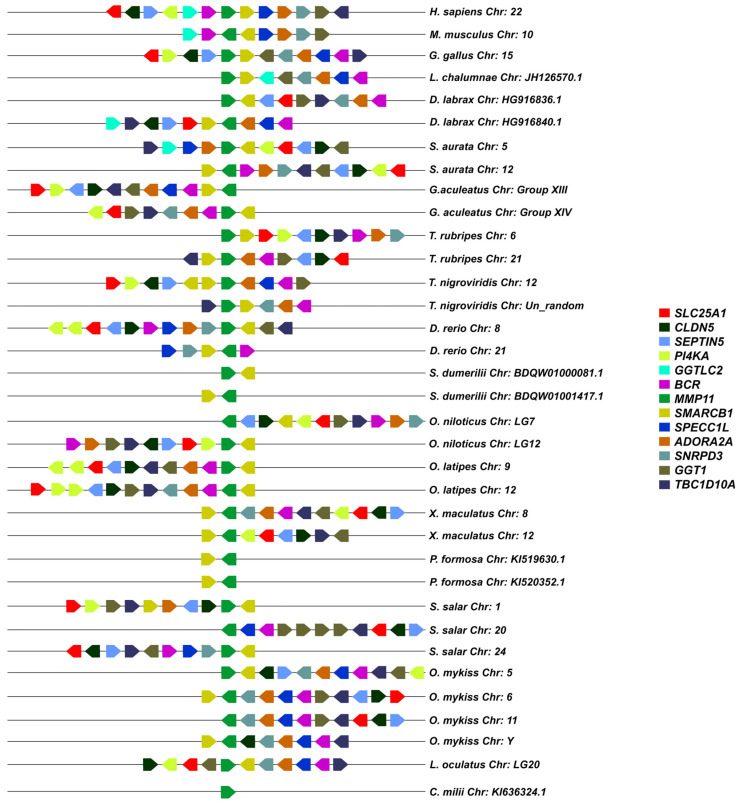
The micro-synteny of *mmp11* is visually depicted in the figure, where colored arrows denote homologous genes, and the arrowhead signifies the direction of predicted gene transcription. Horizontal lines represent chromosome fragments from the included species. Distances between genes or gene length are not drawn to scale.

A macro- and micro-synteny approach was employed to investigate whether the gene paralogs of *mmp11* and *mmp13* originated from genome duplications. Genome duplications typically exhibit characteristic synteny, as blocks of genes within a genome may be found on two distinct chromosomes. The macro-synteny analysis unveiled shared syntenic regions within the genomes of both gilthead sea bream and European seabass MMP paralogs (Supplementary Figures S5 and S6). Furthermore, employing McScan-X tools for collinearity block construction and the “duplicate gene classifier” led to the categorization of paralogs into four categories, as delineated by Wang et al. (2012) [30] (Table 3). In addition, micro-synteny analysis was conducted using Biomart data for all species used for tree reconstructions. The comparisons, extending six (6) genes upstream and downstream of the gene of interest, revealed distinct instances of genes that appear to be duplicated across various teleosts. The micro-synteny analysis revealed high conservation of synteny across species for the *mmp13* gene (Figure 7). In contrast, for the *mmp11* gene, although the majority of the upstream and downstream genes were present across all species, they were not arranged in conserved blocks (Figure 8).

### 3.3. Comparative Protein Structure Analysis

**Figure 9:**
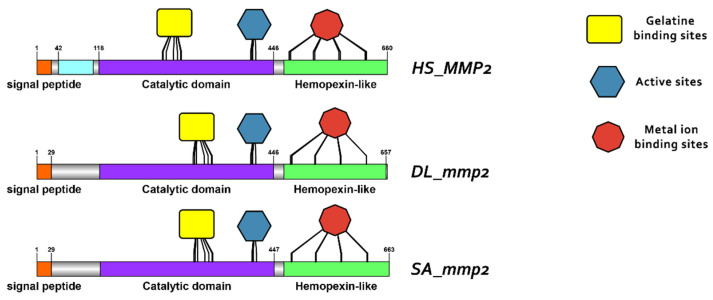
Domain architecture of MMP2 proteins in human (*H. sapiens*), gilthead sea bream (*S. aurata*) and European sea bass (*D. labrax*), including the signal peptide (orange), proteoglycan binding site (PGBD) (light blue), catalytic domain (purple), hemopexin-like domain (light green), gelatine binding sites (yellow), metal ion-binding site (red), and the active sites (dark blue) of the enzyme. The PGBD domain could not be identified in gilthead sea bream (*S. aurata*) and European sea bass (*D. labrax*).

**Figure 10:**
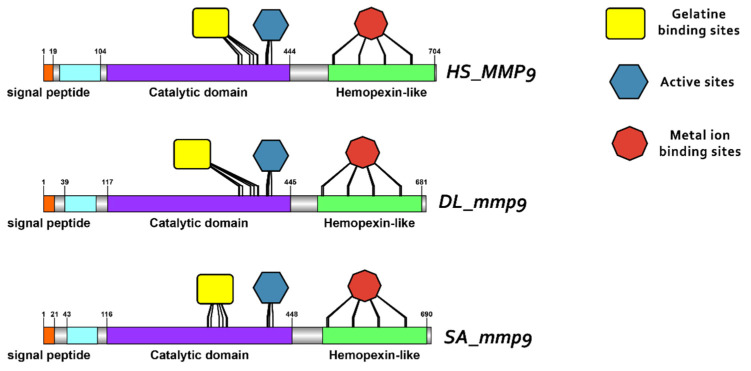
Domain architecture of MMP9 proteins in human, gilthead sea bream and European seabass, including the signal peptide (orange), proteoglycan binding site (PGBD) (light blue), catalytic domain (purple), hemopexin-like domain (light green), gelatine binding sites (yellow), metal ion-binding site (red), and the active sites (dark blue) of the enzyme.

**Figure 11:**
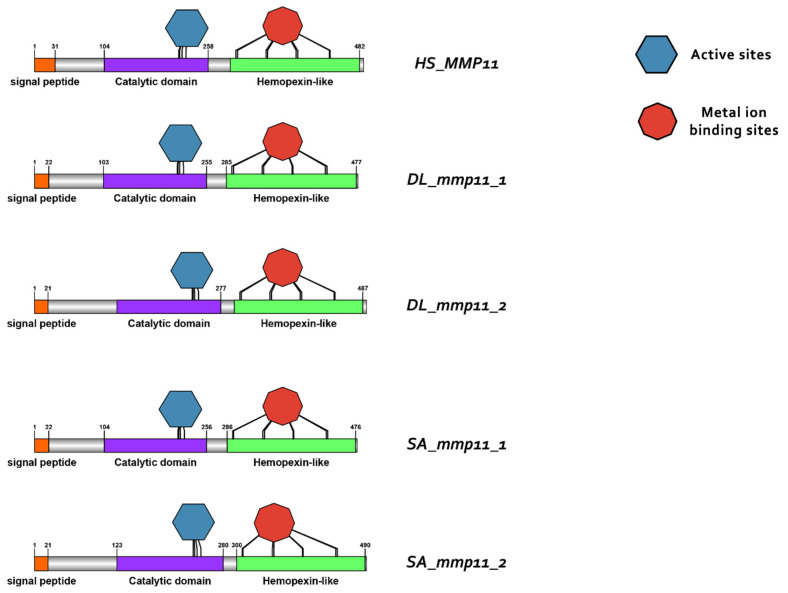
Domain architecture of MMP11 proteins in human (*H. sapiens*), gilthead sea bream (*S. aurata*) and European sea bass (*D. labrax*), including the signal peptide (orange), catalytic domain (purple), hemopexin-like domain (light green), metal ion-binding site (red), and the active sites (dark blue) of the enzyme.

**Figure 12:**
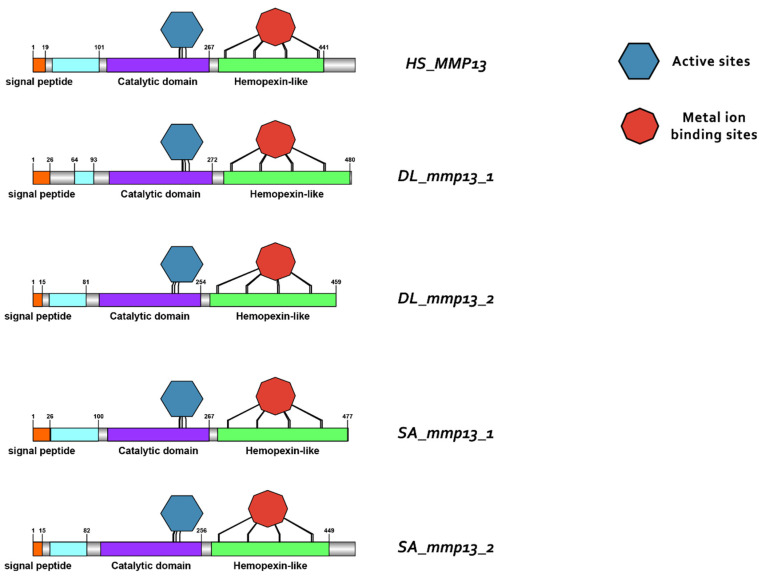
Domain architecture of MMP13 proteins in human (*H. sapiens*), gilthead sea bream (*S. aurata*) and European sea bass (*D. labrax*), including the signal peptide (orange), proteoglycan binding site (PGBD) (light blue), catalytic domain (purple), hemopexin-like domain (light green), metal ion-binding site (red), and the active sites (dark blue) of the enzyme.

**Figure 13:**
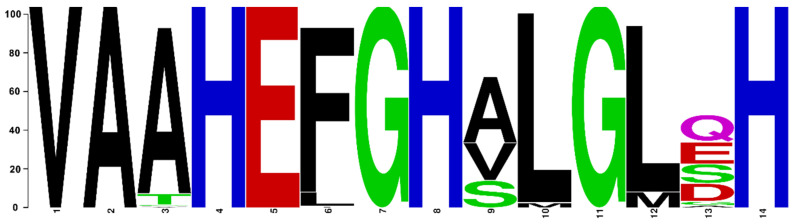
Percentage of conservation in each position of the highly conserved sequence VAAHEXGHXXXXGXXH in vertebrates.

Protein reconstruction was performed using the IBS illustrator, revealing the structural domains of MMPs in humans (*H. sapiens*), gilthead sea bream (*S. aurata*) and European sea bass (*D. labrax*). Notably, all metalloproteases exhibited several structural similarities, including the presence of a signal peptide, facilitating their secretion as proenzymes. In addition, the copies of the duplicated genes *mmp11* and *mmp13* displayed a high degree of conservation, featuring comparable-sized catalytic and regulatory domains. Moreover, both copies retained intact active sites and metal ion-binding sites, affirming their catalytic activity (Figure 9, Figure 10, Figure 11 and Figure 12). It is important to highlight the highly conserved sequence (VAAHEXGHXXXXGXXH) observed in the active site of the catalytic domain of all metalloproteases under study in both gilthead sea bream (*S. aurata*) and European sea bass (*D. labrax*). Generalizing this finding, the same sequence was aligned and retrieved from all species and all metalloproteases under study, including hagfish (*Eptatretus burgeri*) and lamprey (*Petromyzon marinus*). The percentage of conservation remains high throughout vertebrate evolution (Figure 13).

### 3.4. Gene Expression Analysis

#### 3.4.1. Developmental Expression Patterns

Transcriptome analysis revealed three distinct expression patterns of *mmp11* and *mmp13* paralogs, both across different developmental stages and within individual stages in both species: (i) paralogs exhibited comparable expression levels, (ii) one paralog was overexpressed in comparison with its copy, indicative of differential regulatory mechanisms influencing individual expression patterns, and (iii) paralogs exhibited stage-specific expression, implying a nuanced regulation during ontogeny. Genes with fewer than 20 reads were characterized as not detectable.

**Figure 14:**
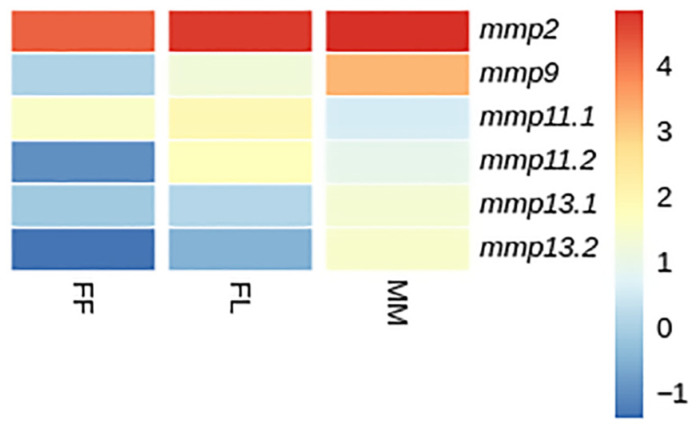
Heatmap of gene expression of *mmps* genes in gilthead sea bream (*S. aurata*) between developmental stages. FF: first feeding; FL: flexion of notochord; MM: mid-metamorphosis. Expression levels are expressed as log_2_CPM (counts per million).

**Figure 15:**
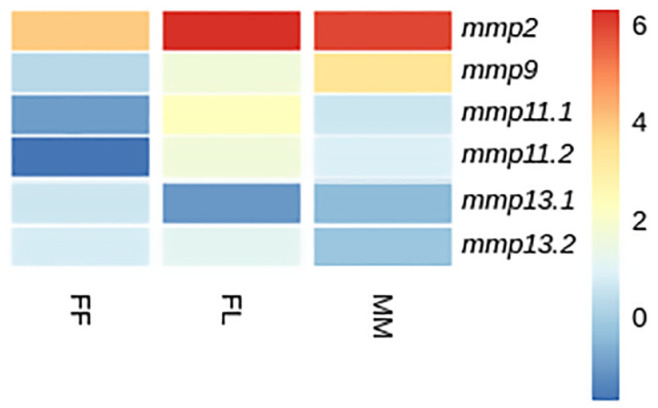
Heatmap of gene expression of *mmps* genes in European sea bass (*D. labrax*) between developmental stages. FF: first feeding; FL: flexion of notochord; MM: mid-metamorphosis. Expression levels are expressed as log_2_CPM (counts per million).

While *mmp11* exhibited a similar expression pattern in both species across and within developmental stages, the expression patterns of *mmp13* varied subtly. Specifically, *mmp13.2* demonstrated expression not detectable above threshold in the FF stage in gilthead sea bream, whereas in European sea bass, the *mmp13.2* paralog exhibited expression not detectable above threshold in the FL stage. This nuanced divergence *mmp13* expression underlines the intricacies of regulatory mechanisms governing specific paralogs and highlights subtle variations in their stage specific/temporal expression profiles between the two species. Single-copy genes, i.e., *mmp2* and *mmp9,* exhibited a similar ontogenetic expression pattern in both species. This concurrence implies a conserved regulatory mechanism for these genes, elucidating a shared ontogenetic regulation in both species (Figure 14 and Figure 15).

#### 3.4.2. Tissue Expression Patterns

**Figure 16:**
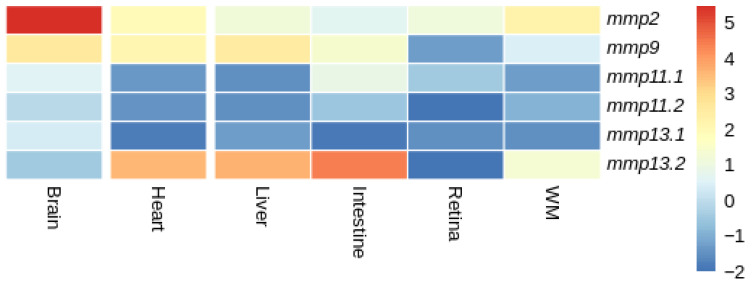
Heatmap of gene expression of *mmps* genes in gilthead sea bream (*S. aurata*) across tissues. Expression levels are expressed as log_2_CPM (counts per million).

**Figure 17:**
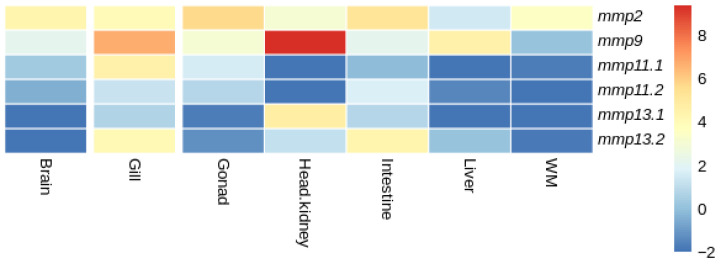
Heatmap of gene expression of *mmps* genes in European sea bass (*D. labrax*) bream across tissues. Expression levels are expressed as log_2_CPM (counts per million).

Our investigation to elucidate the expression patterns of MMPs paralogs extended to the analysis of publicly available transcriptome sequencing data, from adult tissues for both species. Within the constraints of the available data, we acknowledge that not all tissues were included in the study to allow for a more comprehensive analysis. As with developmental expression patterns, the same three patterns of expression were observed (Figure 16 and Figure 17).

In gilthead sea bream (*S. aurata*), both *mmp11* paralogs were expressed at similarly low levels in the heart, liver and white muscle. Their expression was low in all tissues, yet it differentiated in the intestine, retina and brain with *mmp11.1* overexpressed in comparison with *mmp11.2* (Figure 16). Similarly, *mmp13.2* was overexpressed in comparison with *mmp13.1* in the liver, intestine, heart and white muscle, while it exhibited expression not detectable above threshold in the retina.

In European sea bass (*D. labrax*), expression not detectable above threshold was observed for both *mmp11* paralogs in head kidney, while expression not detectable above threshold was observed for *mmp11.1* and *mmp11.2* in the liver and white muscle, respectively. In the gills, gonads, intestine, and liver the two paralogues were differentially expressed; the expression of *mmp11.1* was higher in the gills and gonad, while *mmp11.2* was expressed more in the intestine and liver. Expression not detectable above threshold was observed for both *mmp13* paralogs in the brain, while expression not detectable above threshold was observed for the *mmp13.1* paralog in the liver and white muscle (Figure 17).

When comparing the expression patterns of MMP paralogs between gilthead sea bream (*S. aurata*) and European sea bass (*D. labrax*), distinct differences were observed within common tissues. In particular, *mmp11.1* in European sea bass (*D. labrax*) showed expression not detectable above threshold in the liver, in contrast to the uniform expression observed in gilthead sea bream (*S. aurata*). Similarly, *mmp11.2* was not expressed in the white muscle of European sea bass (*D. labrax*) while both paralogs were expressed in gilthead sea bream (*S. aurata*). In European sea bass (*D. labrax*), neither of the two *mmp13* paralogs were expressed in the brain, while in gilthead sea bream (*S. aurata*), both paralogs were expressed, albeit at relatively low levels. Similarly, in European sea bass (*D. labrax*)only one of the two paralogs of *mmp13* was expressed with expression not detectable above threshold being observed for *mmp13.1*, whereas both *mmp13* paralogs were expressed in gilthead sea bream (*S. aurata*). A ubiquitous expression of *mmp2* and *mmp9* that have no paralogs was observed in both species.

## 4. Discussion

### 4.1. Evolutionary Path of Matrix Metalloproteases in Teleosts

Matrix metalloproteases are present across all domains of life, showing remarkable complexity in vertebrates. Phylogenetic studies reveal orthologous genes in *Ciona intestinalis* (Sea vase), an invertebrate of the subphylum *Urochordata* and a close relative of vertebrates, suggesting that MMP evolution predates the vertebrate-urochordate divergence [6]. The intricate diversity within vertebrate MMPs, however, originates from evolutionary events during early vertebrate lineage development, involving extensive duplication of primordial genes. Successive duplications gave rise to the MMP gene family in vertebrates. While teleosts underwent a lineage-specific whole-genome duplication (TSGD), a similar number of genes are found in both humans and teleosts, implying a low retention of gene duplicates in teleosts [39]. The absence of certain human orthologs in teleosts, such as *MMP1* and *MMP8* genes, suggests the specific loss of these genes in the teleost lineage. This could be attributed to various factors, including functional redundancy, as other collagenolytic metalloproteases like *MMP13* are found in two copies [40]. This redundancy may enable teleosts to achieve collagenolytic functions without relying on *MMP1* and *MMP8*. Additionally, distinct biomechanical and physiological demands on connective tissues, such as teleosts lacking the tendon system and having more complex forms of collagen, might have influenced the collagenolytic machinery in teleosts, rendering these metalloproteases redundant [41].

In this study, we systematically identified and characterized matrix metalloproteinase (MMP) genes with collagenolytic activity. Through phylogenetic analysis, we traced the presence of paralogs within the genomes of teleost species, notably *mmp11* and *mmp13*, back to events such as the Teleost-specific genome duplication (TSGD) and the second round of vertebrate whole-genome duplication (2R), unlike *mmp2* and *mmp9* that were found in one copy, with the exception of the Salmonidae that underwent a lineage-specific genome duplication after the TSGD (SsWGD). The findings for *mmp2* and *mmp9*, retaining one copy in teleosts is in agreement with analogous phylogenetic investigations in *Ctenopharyngodon idella* (grass carp) and *Pelteobagrus fulvidraco* (yellow catfish) [42,43]. Also, these genes share a structural similarity across species, with similar domains, suggesting the conservation of the protein across all species [43].

Two paralogs of *mmp11* were identified in all teleosts but the salmonids. Phylogenetic analysis suggests that these paralogs most probably derived from the TSGD, as one copy of the gene is found in non-teleost vertebrates, and the spotted gar (*L. oculatus*) serves as an outgroup of a well-supported duplication node at the base of teleosts. Macro-synteny analysis provided syntenic regions in both chromosomes where *mmp11* resides (Supplementary Figure S1). Micro-synteny analysis for the *mmp11* gene revealed a less conserved landscape across species. The genes were not arranged in conserved blocks and classified from MCScanX tool as “dispersed”, which is not expected from paralogs derived from genome duplications [44,45]. Additionally, the analysis indicated chromosome rearrangements, consistent with the process of rediploidization, after duplication events [9,46,47] (Figure 7). This discrepancy likely reflects post-WGD genome rearrangements, gene relocations, or annotation inconsistencies that disrupt conserved collinearity, leading to misclassification in computational frameworks. Such limitations are well-documented in comparative genomic analyses, particularly for older duplication events where synteny erosion may obscure true duplication origins. Unlike *mmp11*, which showed a lack of conserved synteny, *mmp13* exhibited high synteny conservation, with a conserved gene block across species (Figure 8).

**Figure 18:**
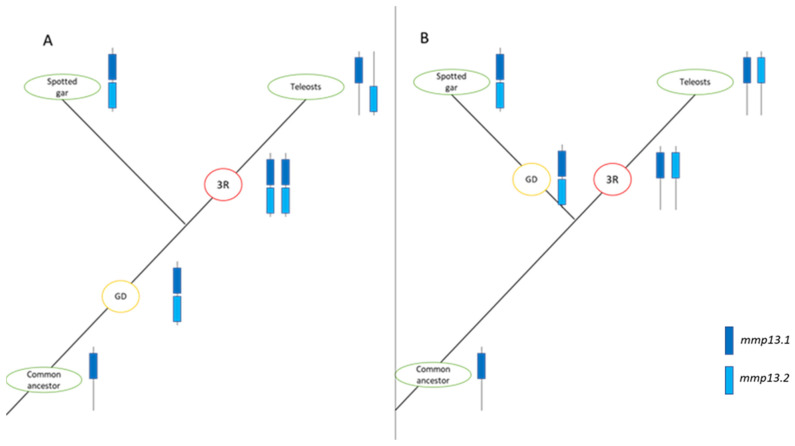
Possible scenarios for the evolutionary pathway of *mmp13* in spotted gar (*L. oculatus*) and Teleosts. *mmp13.1* is represented in dark blue, and *mmp13.2* in light blue. Red circles denote Teleost-Specific Whole-Genome Duplication (TS-WGD or 3R), while orange circles indicate gene or genome duplication (GD). (**A**). In this scenario, duplication occurred in a common ancestor, followed by the conversion of genes resulting from TS-WGD into pseudogenes. (**B**). This scenario suggests an independent gene duplication in the spotted gar (*L. oculatus*) lineage, with the conservation of TS-WGD products in teleosts.

Similarly, in previous studies on the genomes of zebrafish (*D. rerio*) [7], fugu (*T. rubripes*) [48,49], and medaka (*O. latipes*) [50], two copies of *mmp13* were identified, while in Atlantic salmon (*S. salar)* and rainbow trout (*O. mykiss*), four paralogs were identified [51,52]. Although the zebrafish (*D. rerio*) paralog (*mmp13.2*) does not seem to follow the species tree and serves as an outgroup for both spotted gar (*L. oculatus*) and the rest of the teleosts, this could result from poor sequence quality or divergent evolution. Other phylogenetic analyses suggests that divergent evolution is quite common after whole-genome duplication events [53,54]. The presence of two copies of *mmp13* could be interpreted as a balancing counteract to the lack of *mmp1* and *mmp8* through sub-functionalization or gene dosage effect of the two paralogs. Although teleosts underwent the TSGD, the origins of the paralogs of *mmp13* seems more vague since two copies of *mmp13* can also be found in spotted gar, which did not undergo the TSGD [55]. Two possible scenarios may explain this pattern. In the first scenario, a duplication event occurred in the common ancestor of teleosts and spotted gar, followed by the pseudogenization of one or more TSGD-derived *mmp13* copies in teleosts. In the second scenario, the duplication in spotted gar arose independently, while teleosts retained both *mmp13* paralogs that originated from the TSGD. These alternative evolutionary pathways are illustrated in Figure 18. Interestingly, *mmp1* and *mmp8* genes are missing from the spotted gar (*L. oculatus*) genome too, emphasizing the importance of the retention of *mmp13* paralogs. This scenario is further supported by the finding of high sequence identity between the mammals’ genes *MMP1* and *MMP13* (86% amino acid identity) [56].

### 4.2. Conservation of Structure

Protein domain and motifs analysis using the Interpro database highlighted structural similarities among MMPs, emphasizing the conserved nature of key domains for protein function. The catalytic residues of an enzyme comprise the amino acids located in the active center responsible for accelerating the enzyme-catalyzed reaction by lowering the activation energy of the reaction [57]. Evolution tends to strictly conserve the site of the catalytic metal ion. Even small variations that could affect the metal site of a metalloenzyme as MMPs are under strong selection [58]. Functional traits that provide adaptive advantages in each environment are preserved by natural selection. Preserving critical functional domains and residues across species reflect the evolutionary importance of maintaining enzymatic activity and substrate specificity [57,58]. The loss of catalytic residues in enzymes may have been the reason behind lost paralogs of MMPs. The highly conserved sequence (VAAHEXGHXXXXGXXH) in the active site of the catalytic domain of all MMPs under study across vertebrates accentuates the functional significance of this region and underscores the functionality of the proteins.

### 4.3. Expression Analysis

Expression analysis in both tissues and developmental stages of gilthead sea bream (*S. aurata*) and European sea bass (*D. labrax*) unveiled regulatory patterns among the paralogs, with three expression patterns being emerged, indicating potential divergences in regulatory mechanisms. Since these genes are crucial in bone [59,60,61] and tissue remodeling [62] and participate in immune responses [63], they hold great significance in the development of fish larvae. Larvae undergo dramatic changes with tissue growth, bone expansion and the need to survive in a somewhat hostile environment. Through strictly controlled degradation of the extracellular matrix (ECM) by MMPs, tissue is rearranged, facilitating the making of normal structures and ensuring physiological functions [64]. MMPs are also involved in the degradation of bone matrix during bone resorption, a process mediated by osteoclasts; in addition, MMPs help in wound healing and tissue repair, facilitating the breakdown of damaged ECM [65,66,67,68]. In the context of fish development, MMPs play a role in shaping the initial skeletal structures and modulating the ECM, ensuring proper tissue turnover [4]. Furthermore, in scenarios like fin regeneration, MMPs facilitate the remodeling of tissues, showcasing their importance in regenerative processes [69,70].

Studies in zebrafish (*D. rerio*) showed that one of the two paralogs is not detectable during embryogenesis, while the other appears to play a crucial role in hatching [48]. Regarding the role of *mmp13* in bone remodeling in zebrafish (*D. rerio*), there are studies that conclude that *mmp13.1* may be restricted to the endoskeletal part of the fin and the axial skeleton of zebrafish (*D. rerio*), and that *mmp13.2* plays an important role in fin bone formation [71,72,73].

In tissues, *mmp11* and *mmp13* paralogs showed tissue-specific expression in both species, with inter-species differences observed. Drawing data from expression atlas (https://www.ebi.ac.uk/gxa/home, accessed on 27 January 2024) for mammals such as human (*H. sapiens*) (Illumina body map) and mouse (*M. musculus*) (Fantom5 project) for *MMP11* expression across tissues, we can see a ubiquitous expression. On the contrary, in gilthead sea bream (*S. aurata*) and European sea bass (*D. labrax*) the expression of the paralogs of *mmp11* is tissue-specific (Figure 16 and Figure 17).

The expression of *MMP13* is tissue-specific in humans, with only a few tissues expressing this gene. On the contrary, in rodents like mice, and rabbits [74], the expression is extended to more tissues. *MMP1,* absent in teleosts, shares high homology with *MMP13* in mammals and is ubiquitous in humans. Interestingly, there is also a rodent-specific duplication of *MMP1*, though no relevant expression data is available [56].

On the other hand, at least one *mmp13* paralog was expressed in almost all tissues studied in gilthead sea bream (*S. aurata*) and European sea bass (*D. labrax*) except of the brain tissue in European sea bass (*D. labrax*). A study conducted by Castillo-Briceno and colleagues in different tissues of adult samples of gilthead sea bream (*S. aurata*) confirmed the expression of *mmp13.2* across all tissues studied, although it should be noted that only that paralog was taken into consideration, probably due to shortcomings of the databases at the time [75].

### 4.4. Fate of Paralogs

The complex genetic interaction network of dispensable WGD paralogs provides insight into the long-standing question of why paralogs with overlapping functions are retained on an evolutionary time scale [76]. Duplicated genes are initially redundant in function, meaning duplicates have the same function, with none or minor phenotypic consequences in the case that one copy is eliminated [18]. The remaining paralogous gene is sufficient to compensate for the loss of the second paralog. Therefore, redundant duplicates provide an ideal source of genetic material that can be used as the origin of new genes [17]. Paralogs can follow different fates after the initial duplication. Paralogs can be maintained or eliminated through selective pressure during evolution. In general, the loss of an extra copy is more frequent than the maintenance of a functionally redundant copy [9,18,49,77] and that appears to be the case of *mmp2* and *mmp9* paralogs where the subsequent rediploidization process after a WGD led the paralogs from the TSGD being lost [78]. Phylogenetic analysis of the *mmp2* and *mmp9* genes revealed a conserved single-copy presence across all teleosts, except within the Salmonidae branch, where two copies were observed, consistent with the fourth round of genome duplication specific to this lineage.

In circumstances where the duplicated gene is retained, mutations may arise in the coding or regulatory sequence at different positions of each duplicate, leading to changes in the coding sequence and the protein structure, or the spatiotemporal regulation of the gene expression [18,49,79,80,81,82,83]. While transcriptome analysis provides insight into the expression patterns of the retained paralogs of *mmp11* and *mmp13* in gilthead sea bream (*S. aurata*) and European sea bass (*D. labrax*), determining their exact evolutionary fate requires more direct functional assays.

Considering conserved catalytic domain sequences between paralogs, we hypothesize that the catalytic function of both paralogs is retained. The identification of distinct expression levels and patterns either between developmental stages or across tissues suggests a degree of regulatory sub-functionalization. The nuanced variations in expression may be ascribed to mutations in regulatory elements or the acquisition of specific cis-regulatory motifs, handing a differential response of the paralogs. However, since *mmp11* is expressed across tissues in mammals and in teleosts pose a tissue-specific expression and *mmp13* seems to have broader function in teleosts, compensating probably for the loss of *MMP1*, the regulatory neo-functionalization cannot be excluded.

### 4.5. Limitations of the Study

While this study provides biologically meaningful insights into the evolution and expression of collagenolytic *MMPs* in teleosts and advances our current knowledge, certain methodological limitations should be acknowledged.

RNA-seq analyses for developmental stages were conducted on pooled samples without biological replicates, limiting the statistical power for differential expression. Similarly, publicly available transcriptomic datasets used for tissue expression analysis also lacked replicates, constraining the robustness of statistical comparisons. Although all analyses followed best practices for non-replicated datasets (edgeR), and expression patterns were consistent across stages and species and biologically meaningful, the findings should be interpreted with caution and validated experimentally.

Additionally, functional predictions of protein domains are relied on in silico tools, which are subject to limitations such as annotation quality and algorithmic assumptions. Some undetected domains may reflect technical artifacts rather than true biological absence. As such, domain-based inferences should be considered preliminary until supported by experimental validation.

## 5. Conclusions

This study tested the hypothesis that matrix metalloprotease (MMP) paralogs in teleosts, specifically *mmp11* and *mmp13*, have undergone regulatory divergence following major duplication events such as the second round of vertebrate whole-genome duplication (2R) and the teleost-specific genome duplication (TSGD). Phylogenetic and synteny analyses confirmed a teleost-specific expansion of collagenolytic MMPs and revealed the absence of *mmp1*, a major interstitial collagenase present in mammals. Our data suggests that this loss may have driven compensatory functional specialization of *mmp13a* and *mmp13b*, which display differential and tissue-specific transcriptional regulation in gilthead sea bream (*S. aurata*) and European sea bass (*D. labrax*). Through comparative phylogenetic analysis and transcriptomic profiling, we demonstrate that these paralogs are not only retained but have acquired distinct expression patterns across tissues and developmental stages. This divergence, despite conservation of key catalytic residues, supports a model of regulatory sub-functionalization and potentially regulatory neo-functionalization. These findings provide mechanistic insight into how gene duplicates evolve under selective pressures to perform complementary roles in processes such as tissue remodeling, immune modulation, and skeletal development. Future functional studies, including gene knockout or knockdown experiments, are essential to validate the physiological relevance of these paralogs and to further elucidate the consequences of their differential regulation. Overall, this work offers a refined perspective on how genome duplication events contribute to the diversification and specialization of gene functions, enhancing our understanding of the molecular evolution of vertebrate genomes.

## Figures and Tables

**Table 1:** Species selected for the phylogenetic analysis and the accession numbers of each species genome. *: Stickleback and Tetraodon hadn’t any accession number and we report the Ensembl Assembly ID.

Species	Representative of	Genome
*Sparus aurata* (Gilthead sea bream)	Teleosts	GCA_900880675.1
*Dicentrarchus labrax* (European sea bass)	Teleosts	GCA_000689215.1
*Seriola dumerilii* (Greater amberjack)	Teleosts	GCA_002260705.1
*Gasterosteus aculeatus* (Stickleback)	Teleosts	BROAD S1 *
*Takifugu rubripes* (Fugu)	Teleosts	GCA_901000725.2
*Tetraodon nigroviridis* (Tetraodon)	Teleosts	TETRAODON 8.0 *
*Oreochromis niloticus* (Nile tilapia)	Teleosts	GCA_001858045.3
*Oryzias latipes* (Japanese medaka)	Teleosts	GCA_002234675.1
*Poecilia formosa* (Amazon molly)	Teleosts	GCA_000485575.1
*Xiphophorus maculatus* (Monterrey platyfish)	Teleosts	GCA_001444195.1
*Danio rerio* (Zebrafish)	Teleosts	GCA_000002035.4
*Salmo salar* (Atlantic salmon)	Teleosts	GCA_905237065.2
*Oncorhynchus mykiss* (Rainbow trout)	Teleosts	GCA_013265735.3
*Lepisosteus oculatus* (Spotted gar)	*Actinopterygii*	GCA_000242695.1
*Latimeria chalumnae* (Coelacanth)	*Sarcopterygii*	GCA_000225785.1
*Homo sapiens* (Human)	*Sarcopterygii*	GCA_000001405.28
*Mus musculus* (Common mouse)	*Sarcopterygii*	GCA_000001635.9
*Gallus gallus* (Chicken)	*Sarcopterygii*	GCA_000002315.5
*Xenopus tropicalis* (Tropical clawed frog)	*Sarcopterygii*	GCA_000004195.3
*Callorhinchus milii* (Elephant shark)	*Chondrichthyes*	GCA_000165045.2

**Table 2:** Correspondence between the masked gene names and the according Ensembl gene IDs in both gilthead sea bream and European sea bass.

Gene	Gilthead Sea Bream (*S. aurata*)	European Sea Bass (*D. labrax*)
*mmp2*	ENSSAUG00010014278	ENSDLAG00005001814
*mmp9*	ENSSAUG00010026521	ENSDLAG00005018682
*mmp11.1*	ENSSAUG00010019504	ENSDLAG00005011760
*mmp11.2*	ENSSAUG00010022437	ENSDLAG00005009945
*mmp13.1*	ENSSAUG00010014101	ENSDLAG00005008130
*mmp13.2*	ENSSAUG00010010684	ENSDLAG00005008348

**Table 3:** Duplicate genes classification in gilthead sea bream and European sea bass using McScan-X tools.

Gene Name	Gilthead Sea Bream	European Sea Bass
*mmp11*	Dispersed	Dispersed
*mmp13*	WGD/segmental	WGD/segmental

## Data Availability

All consensus sequences were retrieved from the Short Read archive (SRA) under Accession No (*D. labrax*: PRJNA1050410 and *S. aurata*: PRJNA1050571).

## Supplementary Materials

The following supporting information can be downloaded at https://www.mdpi.com/article/10.3390/ani15223270/s1, Figure S1. Exon–intron structure of *mmp2* genes in *Dicentrarchus labrax* (DL), *Sparus aurata* (SA), and *Homo sapiens* (HS). Schematic representation of the gene architecture of *mmp2* orthologs based on genomic and coding sequences. Coding sequences (CDS) are shown as purple boxes, introns as black lines, and untranslated regions (UTRs or upstream/downstream regions) as yellow boxes. The direction of transcription proceeds from 5′ to 3′. Scale bars indicate relative distances; Figure S2. Exon–intron structure of *mmp9* genes in *Dicentrarchus labrax* (DL), *Sparus aurata* (SA), and *Homo sapiens* (HS). Schematic representation of the gene architecture of *mmp9* orthologs based on genomic and coding sequences. Coding sequences (CDS) are shown as purple boxes, introns as black lines, and untranslated regions (UTRs or upstream/downstream regions) as yellow boxes. The direction of transcription proceeds from 5′ to 3′. Scale bars indicate relative distances; Figure S3. Exon–intron structure of *mmp11* genes in *Dicentrarchus labrax* (DL), *Sparus aurata* (SA), and *Homo sapiens* (HS). Schematic representation of the gene architecture of *mmp11* orthologs based on genomic and coding sequences. Coding sequences (CDS) are shown as purple boxes, introns as black lines, and untranslated regions (UTRs or upstream/downstream regions) as yellow boxes. The direction of transcription proceeds from 5′ to 3′. Scale bars indicate relative distances; Figure S4. Exon–intron structure of *mmp13* genes in *Dicentrarchus labrax* (DL), *Sparus aurata* (SA), and *Homo sapiens* (HS). Schematic representation of the gene architecture of *mmp13* orthologs based on genomic and coding sequences. Coding sequences (CDS) are shown as purple boxes, introns as black lines, and untranslated regions (UTRs or upstream/downstream regions) as yellow boxes. The direction of transcription proceeds from 5′ to 3′. Scale bars indicate relative distances; Figure S5. Circos plot depicting the paralogue relationships between gilthead sea bream genes. Duplicated gene relationships between gilthead sea bream chromosomes are represented by inner lines; Figure S6. Circos plot depicting the paralogue relationships between European sea bass genes. Duplicated gene relationships between European sea bass chromosomes are represented by inner lines. Table S1. Public RNAseq data from various tissues in both *S. aurata* and *D. labrax*. Missing tissue for each species is reported with “-”.
