# Supplementary material for: Evolutionary Dynamics of Matrix Metalloproteases with Collagenolytic Activity in Teleosts
Source: Animals (Basel). 2025 Nov 12;15(22):3270. doi: 10.3390/ani15223270 (PMC12649390; doi:10.3390/ani15223270)
Supplement: Supplementary file 1 [file animals-15-03270-s001.zip › Supplementary Figures.pdf]

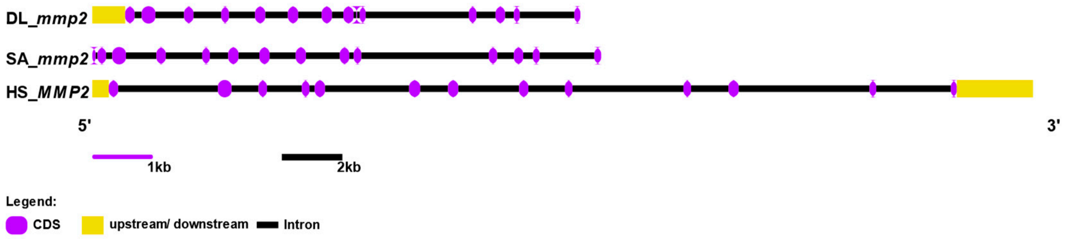

Supplementary Figure S1. Exon–intron structure of *mmp2* genes in *Dicentrarchus labrax* (DL), *Sparus aurata* (SA), and *Homo sapiens* (HS). Schematic representation of the gene architecture of *mmp2* orthologs based on genomic and coding sequences. Coding sequences (CDS) are shown as purple boxes, introns as black lines, and untranslated regions (UTRs or upstream/downstream regions) as yellow boxes. The direction of transcription proceeds from 5' to 3'. Scale bars indicate relative distances.

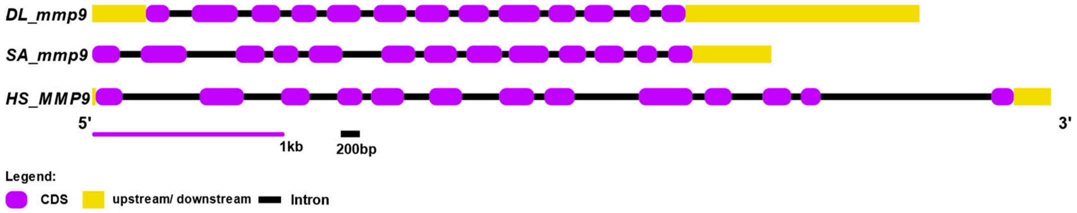

Supplementary Figure S2. Exon–intron structure of *mmp9* genes in *Dicentrarchus labrax* (DL), *Sparus aurata* (SA), and *Homo sapiens* (HS). Schematic representation of the gene architecture of *mmp9* orthologs based on genomic and coding sequences. Coding sequences (CDS) are shown as purple boxes, introns as black lines, and untranslated regions (UTRs or upstream/downstream regions) as yellow boxes. The direction of transcription proceeds from 5' to 3'. Scale bars indicate relative distances.

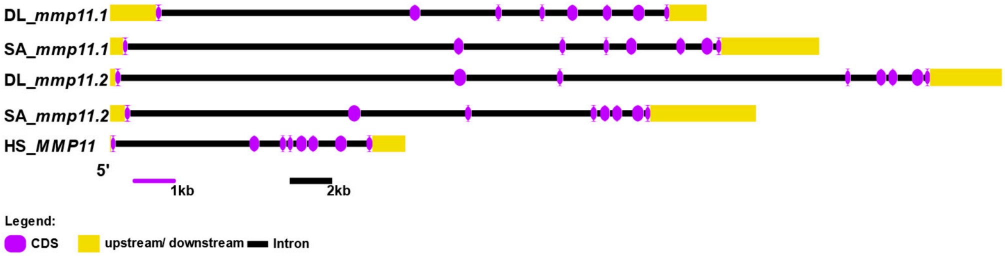

Supplementary Figure S3. Exon–intron structure of *mmp11* genes in *Dicentrarchus labrax* (DL), *Sparus aurata* (SA), and *Homo sapiens* (HS). Schematic representation of the gene architecture of *mmp11* orthologs based on genomic and coding sequences. Coding sequences (CDS) are shown as purple boxes, introns as black lines, and untranslated regions (UTRs or upstream/downstream regions) as yellow boxes. The direction of transcription proceeds from 5' to 3'. Scale bars indicate relative distances.

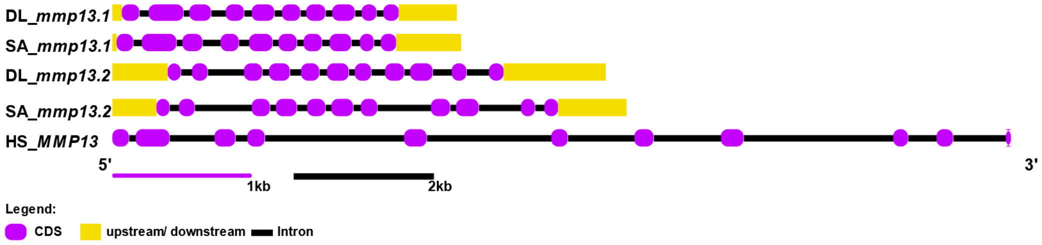

Supplementary Figure S4. Exon–intron structure of *mmp13* genes in *Dicentrarchus labrax* (DL), *Sparus aurata* (SA), and *Homo sapiens* (HS). Schematic representation of the gene architecture of *mmp13* orthologs based on genomic and coding sequences. Coding sequences (CDS) are shown as purple boxes, introns as black lines, and untranslated regions (UTRs or upstream/downstream regions) as yellow boxes. The direction of transcription proceeds from 5' to 3'. Scale bars indicate relative distances.

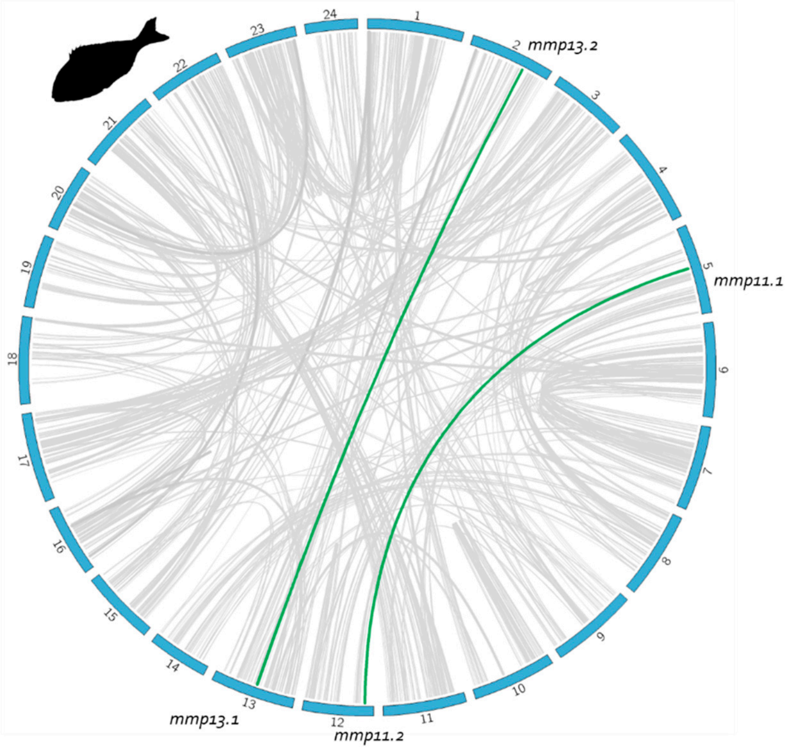

Supplementary Figure S5. Circos plot depicting the paralogue relationships between gilthead sea bream genes. Duplicated gene relationships between gilthead sea bream chromosomes are represented by inner lines.

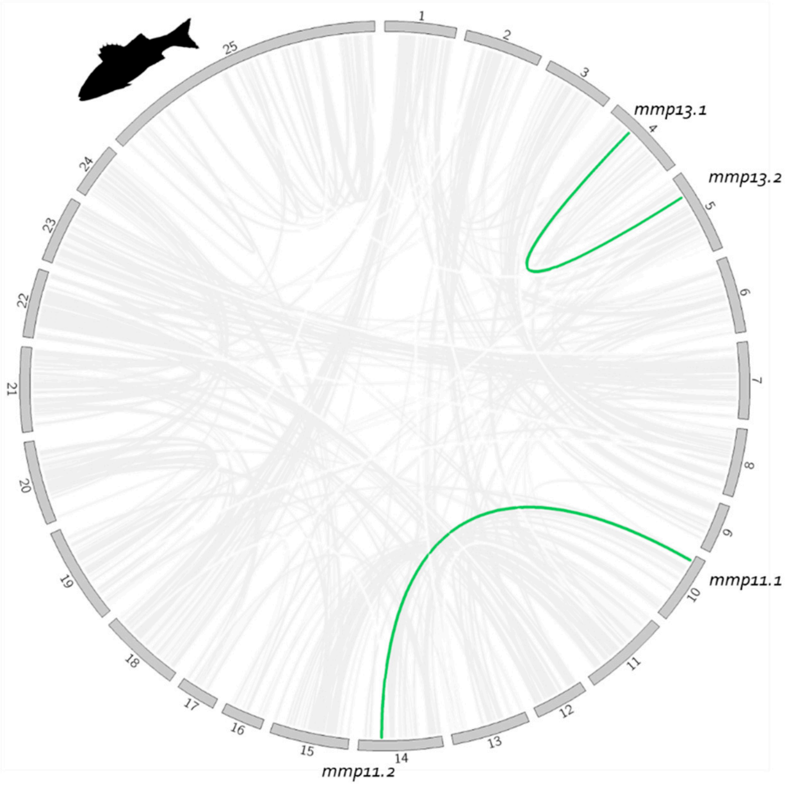

Supplementary Figure S6. Circos plot depicting the paralogue relationships between European sea bass genes. Duplicated gene relationships between European sea bass chromosomes are represented by inner lines.
