# Supplementary material for: Evolutionary Dynamics of Matrix Metalloproteases with Collagenolytic Activity in Teleosts
Source: Animals (Basel). 2025 Nov 12;15(22):3270. doi: 10.3390/ani15223270 (PMC12649390; doi:10.3390/ani15223270)
Supplement: Supplementary file 1 [file animals-15-03270-s001.zip › Supplementary Table S1.pdf]

Supplementary Table S1. Public RNAseq data from various tissues in both *S. aurata* and *D. labrax.* Missing tissue for each species is reported with "- "

Tissue	*S. aurata*	*D. labrax*	Library Type	Instrument
Brain	SRR6237496	ERR9715603	PE 150 bp	Illumina Hiseq
Gill	-	ERR9715604	PE 150 bp	Illumina Hiseq
Gonad	-	ERR9715605	PE 150 bp	Illumina Hiseq
Head kidney	-	ERR9715606	PE 150 bp	Illumina Hiseq
Intestine	SRR6237498	ERR9715623	PE 150 bp	Illumina Hiseq
Liver	SRR6237500	ERR9715614	PE 150 bp	Illumina Hiseq
White muscle	SRR6237499	ERR9715622	PE 150 bp	Illumina Hiseq
Heart	SRR6237497	-	PE 150 bp	Illumina Hiseq
Retina	SRR8242400	-	PE 150 bp	Illumina Hiseq
